# Evidence of Individual Superspin Relaxation in Diluted Fe_3_O_4_/Hexane Ferrofluids

**DOI:** 10.3390/ma16134850

**Published:** 2023-07-06

**Authors:** Cristian E. Botez, Zachary Mussslewhite

**Affiliations:** 1 Department of Physics and Astronomy, University of Texas at San Antonio, 1 UTSA Circle, San Antonio, TX 78249, USA; zachary.musslewhite@utsa.edu; 2 Department of Physics, University of Texas at El Paso, 500 W. University Avenue, El Paso, TX 79968, USA

**Keywords:** magnetic nanoparticles, superspin relaxation, ac susceptibility

## Abstract

We used dc magnetization and ac susceptibility to investigate the magnetic relaxation of ferrofluids made of 8 nm average-diameter Fe_3_O_4_ nanoparticles dispersed in hexane. Samples of different concentrations (δ) spanning two orders of magnitude ranging from 0.66 to 0.005 mg (Fe_3_O_4_)/mL (hexane) were used to vary the interparticle interaction strength. Our data reveal a critical concentration, δ_c_ = 0.02 mg/mL, below which the ferrofluid behaves like an ideal nanoparticle ensemble where the superspins relax individually according to a Néel–Brown activation law $\tau (T)\;=\tau_{0}\exp\left(\frac{E_{B}}{k_{B}T}\right)$ with a characteristic time τ_o_ ~10^−9^ s. That is further confirmed by the observed invariance of the relative peak temperature variation per frequency decade $∆=\frac{∆T}{T\cdot ∆log(f)}$, which stays constant at ~0.185 when δ < δ_c_. At higher concentrations, between 0.02 and 0.66 mg/mL, we found that Δ exhibits a monotonic increase with the inverse concentration, $\frac{1}{\delta }$, and the collective superspin dynamics is described by a Vogel–Fulcher law, $\tau (T)\;=\tau_{0}\exp\left[\frac{E_{B}}{k_{B}\left(T-T_{0}\right)}\right]$. Within this regime, the dipolar interaction strength parameter T_0_ increases from T_0_ = 0 K at δ_c_ = 0.02 mg/mL to T_0_ = 14.7 K at δ = 0.66 mg/mL.

## 1. Introduction

Materials based on magnetic nanoparticle ensembles have recently received a lot of attention due to their potentially transformative applications in magnetic recording [1,2] and biomedicine [3,4,5,6,7]. Much progress has been made in terms of tuning the magnetic response of these systems by adjusting their average size, size distribution, and chemical composition [8,9,10,11,12]. Yet, few studies have been aimed at systematically studying the effect of interparticle dipolar interactions on the system’s magnetic behavior [13,14,15]. This type of investigation is important, particularly when carried out using experimental techniques such as ac susceptibility that can reveal quantitative information on the nanoparticles’ superspin dynamics. Understanding and eventually controlling this behavior is critical for high-density magnetic recording applications where each superspin represents a separate recording bit that needs to relax independently in order to ensure the proper functionality of the recording device [16].

Several models are available to analyze data and gauge the effect of dipolar interactions on the superspin dynamics of a nanoparticle ensemble. The Néel–Brown activation law, for example, describes the temperature dependence of the relaxation time, τ(T), of an ideal ensemble of non-interacting nanoparticles [17]:

(1) $$\tau (T)\;=\tau_{0}\exp\left(\frac{E_{B}}{k_{B}T}\right)$$

Here, τ_0_ is the characteristic time (related to the attempt frequency by τ_o_ = 1/2πf_o_, E_B_ is the energy barrier to magnetization reversal, and k_B_ is the Boltzmann constant.

Equation (1) also describes the blocking of the superspins that occurs upon cooling below the blocking temperature:

(2) $$T_{B}=\frac{E_{B}}{k_{B}\ln(\tau /\tau_{0})}$$

For interacting nanoparticles, Shtrikman and Wohlfarth have developed another model based on a Vogel–Fulcher activation law [18]:

(3) $$\tau (T)\;=\tau_{0}\exp\left(\frac{E_{B}}{k_{B}\left(T-T_{0}\right)}\right)$$

where T_0_ is a parameter that describes the strength of the interparticle dipolar interactions. Finally, for strongly interacting nanoparticles, the superspins might not block superparamagnetically upon cooling, but in a collective, spin-glass-like fashion. This so-called superspin glass freezing has been observed by us [19,20] and others [21,22] in several magnetic nanopowders, and is well described by a power law [23]:

(4) $$\tau (T)\;=\tau_{0}{\left[\frac{T}{T_{g}}-1\right]}^{-z\nu }$$

where T_g_ is the critical freezing temperature, and z and ν are dynamic and static exponents, respectively.

Here, we present a study of the effect of interparticle dipolar interactions on the magnetic properties of 8 nm average-diameter Fe_3_O_4_ nanoparticle ensembles. We varied the interparticle interaction strength by using ferrofluids of different concentrations (δ) ranging from 0.66 to 0.005 mg (Fe_3_O_4_)/mL (hexane). We used dc magnetization and ac susceptibility to measure and analyze the magnetic response of the system as a function of δ. Our main finding is a critical concentration, δ_c_ = 0.02 mg/mL, below which the ferrofluid behaves as an ideal ensemble of (non-interacting) magnetic nanoparticles. This is demonstrated by the shift of the out-of-phase ac susceptibility χ″ vs. T|_f_ peak with the observation time $\tau =\frac{1}{2\pi f}$, which is excellently described by the Néel–Brown activation law (Equation (1)). The aforementioned behavior is further confirmed by the behavior of the relative peak temperature variation per frequency decade $∆=\frac{∆T}{T\Delta \cdot log(f)}$, which stays constant at ~0.185 within this low concentration regime (0.02 mg/mL > δ > 0.005 mg/mL). At concentrations above δ_c_, as the interparticle dipolar interactions become stronger, we found a markedly different behavior. Throughout this higher concentration regime (0.66 mg/mL > δ > 0.02 mg/mL), Δ exhibits a monotonic increase with the inverse concentration, $\frac{1}{\delta }$, and the system’s dynamic behavior is well described by a Vogel–Fulcher law (Equation (3)). We used Equation (3) to determine the values of the interaction strength parameter T_o_, and found that it increases from T_0_ = 0 K at δ_c_ = 0.02 mg/mL to T_0_ = 14.7 K at δ = 0.66 mg/mL.

## 2. Materials and Methods

We used seven samples obtained by progressively diluting an as-prepared ferrofluid synthesized by dispersing 8 nm average-diameter, oleic-acid-coated Fe_3_O_4_ nanoparticles in hexane. A total of 10 mg of Fe_3_O_4_ nanopowder was initially dispersed in 15 mL of hexane to prepare a ferrofluid of density 0.66 mg (Fe_2_O_3_)/mL (hexane). Samples of different concentrations were then made by adding more hexane to the as-prepared ferrofluid to reduce the concentration to 0.16, 0.08, 0.04, 0.02, 0.01, and 0.005 mg/mL. The average size and narrow size distribution of the nanoparticle were confirmed via Transmission Electron Microscopy (TEM) measurements performed on powders obtained from the as-prepared sample upon the evaporation of the carrier fluid (T_F_ = 178 K) All magnetic measurements were carried out at temperatures below the freezing point of hexane using a Quantum Design^®^ Versa Lab (San Diego, CA, USA) equipped with a Vibrating Sample Magnetometer (VSM) and a Quantum Design^®^ Physical Property Measurement System equipped with an AC Measurement System (ACMS). Dc magnetization, M, was measured upon heating from 3 to 150 K in a constant magnetic field of 50 Oe using the field-cooled and zero-field-cooled (FC-ZFC) protocol. An amount of 0.25 mL of ferrofluid was placed inside a polycarbonate capsule at the end of a rod attached to a dc motor. The motor accelerates the sample through a set of pickup coils, and the magnetic moment is measured via electromagnetic induction. The in-phase and out-of-phase components of the ac susceptibility, χ′ and χ″, were then recorded upon heating from 3 K to 100 K. In this case, the polycarbonate capsule containing the ferrofluid is fixed in place, and an alternating magnetic field of amplitude 3 Oe and frequencies spanning a two-order-of-magnitude range between 10^2^ Hz and 10^4^ Hz is applied.

## 3. Results and Discussion

**Figure 1:**
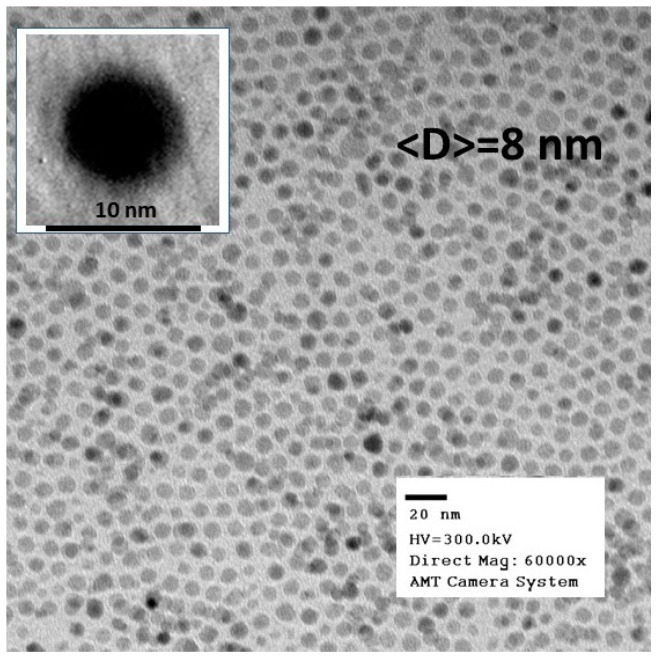
Transmission Electron Microscopy (TEM) images collected on Fe_3_O_4_ powders obtained from the as-prepared δ = 0.66 mg/mL sample.

Figure 1 shows Transmission Electron Microscopy (TEM) images collected on an Fe_3_O_4_ powder obtained via the evaporation of hexane from the δ = 0.66 mg/mL ferrofluid. The TEM data analysis reveals nanoparticles of spherical shape (as demonstrated by the image in the inset) that have a sharp size distribution and an average diameter <D> = 8 nm. All the other ferrofluids used in the present study were made by diluting the as-prepared sample, so we expect them to have similar nanoparticle average sizes, shapes, and size distributions to those of the powder.

**Figure 2:**
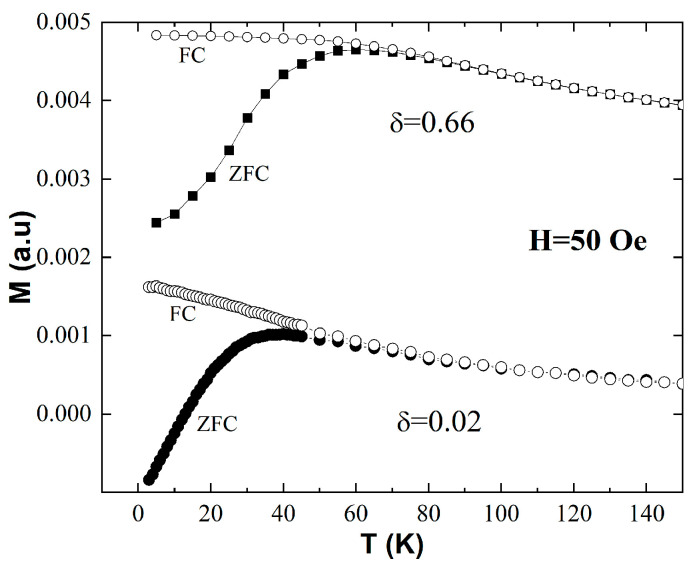
Zero-field-cooled (ZFC) and field-cooled (FC) magnetization vs. temperature curves measured on two samples of different concentrations, δ = 0.66 mg/mL and δ = 0.02 mg/mL.

Figure 2 presents the temperature dependence of the dc magnetization, M vs. T, measured on the δ = 0.66 mg/mL and δ = 0.02 mg/mL samples using the FC-ZFC protocol. All data were collected upon heating from 3 K to 150 K by recording the magnetization, M, measured in an external magnetic field, H = 50 Oe. The empty symbols show the FC curves, where the field was applied at room temperature before cooling down to 3 K, whereas the filled symbols represent the ZFC data, where the sample was cooled in a zero magnetic field and the 50 Oe field was turned on at 3 K. For both samples, the ZFC curves have a peak at T_p_, and the ZFC and FC branches overlap above a given temperature, T_irr_, which marks the onset of magnetic irreversibility. 

This behavior is indicative of a transition from the blocked to the superparamagnetic state of a magnetic nanoparticle ensemble upon heating [24,25]. The most relevant feature of the data in Figure 2 is that both T_p_ and T_irr_ shift to higher values with the increase in δ. Indeed, for δ = 0.02 mg/mL, our data show that T_p_ = 38 K and T_irr_ = 65 K, whereas for δ = 0.66 mg/mL, we found that T_p_ = 60 K and T_irr_ = 80 K. This indicates that the blocked state persists to higher temperatures in the presence of stronger interparticle dipolar interactions. It is important to note, however, that due to the quintessentially dynamic nature of the superparamagnetic behavior, dc magnetization measurements alone cannot provide definite proof of and/or quantitatively describe the details of superspin blocking. For example, the features of the FC-ZFC curves observed here—a peak in the ZFC branch and the onset of magnetic irreversibility upon heating above a temperature threshold—are also signatures of different phenomena, such as a Curie or a Néel transition in the material that the nanoparticles are made of. Consequently, frequency-resolved ac susceptibility measurements carried out at different temperatures are critical to unambiguously establish the superparamagnetic nature of the observed magnetic behavior and to quantitatively describe the microscopic details of the heating-induced superspin unblocking in the systems investigated in this work. These data are presented below.

**Figure 3:**
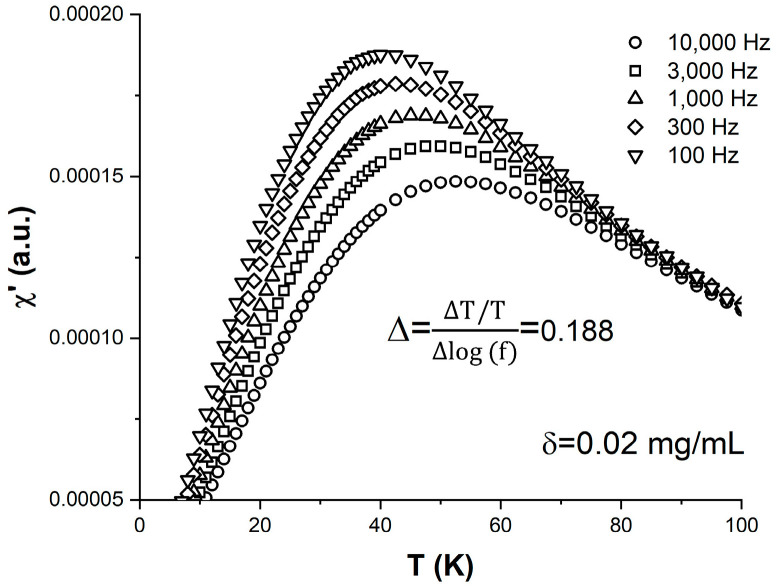
Temperature dependence of the in-phase component of the ac susceptibility χ′ measured on the δ = 0.02 mg/mL sample at different frequencies: 100 Hz (inverted triangles), 300 Hz (diamonds), 1000 Hz (upright triangles), 3000 Hz (squares), and 10,000 Hz (circles).

The temperature behavior of the in-phase component of the ac susceptibility, χ′, measured on the δ = 0.02 mg/mL sample is shown in Figure 3. The five curves correspond to different measurement frequencies f (or observation times $\tau =\frac{1}{2\pi f}$) that span a broad, two-order-of-magnitude range between 100 and 10,000 Hz. All the χ′ vs. T|_f_ curves exhibit a robust peak that shifts towards higher temperatures with the increase in the measurement frequency/decrease in the observation time. The magnitude of the shift is often described by the so-called relative peak temperature variation per frequency decade $∆=\frac{∆T}{T\cdot ∆log(f)}$. From the data in Figure 3, we found that Δ = 0.188 for the δ = 0.02 mg/mL sample. It is known that Δ contains information about the interparticle dipolar interaction strength within a magnetic nanoparticle ensemble, and its value has previously been used to distinguish between systems that undergo superspin blocking upon cooling in the presence of relatively week interparticle interactions and their strong-interaction counterparts where the superspins freeze in a spin-glass-like fashion [26,27]. Values of Δ below 0.05 typically indicate a superspin glass transition, whereas values above 0.1 indicate a superspin blocking–unblocking superparamagnetic transition. For superparamagnetic nanoparticle ensembles, Δ increases with the decrease in the interparticle interactions, which in our case happens upon dilution. This occurs as the blocking temperature increases more per frequency decade for weaker interactions, according to Equation (3). Once the magnetic dipolar interactions become negligible, and the superparamagnetic blocking is described by Equation (1), further dilution below δ_c_ should not affect the value of Δ. One of our goals here is to establish a relationship between Δ and quantities directly linked to the strength of dipolar interactions, such as the ferrofluid density and the average interparticle distance.

**Figure 4:**
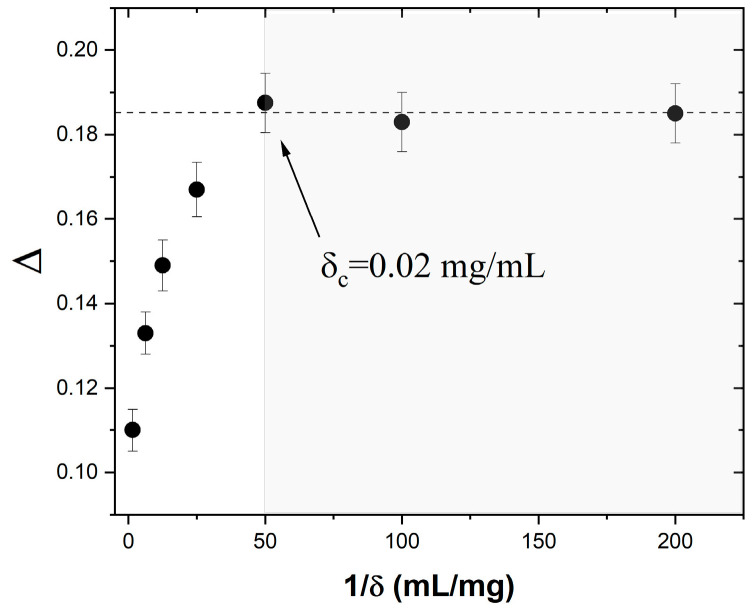
Dependence of the relative peak temperature variation per frequency decade $∆=\frac{∆T}{T\cdot \Delta log(f)}$ on the inverse concentration $\frac{1}{\delta }$.

To that end, we carried out ac susceptibility measurements and data analyses similar to those in Figure 3 on six other ferrofluids of different concentrations: δ = 0.66, 0.16, 0.08, 0.04, 0.01, and 0.005 mg (Fe_3_O_4_)/mL (hexane). For each of them, we determined the relative peak temperature variation per frequency decade, Δ, from the shift with the frequency of the χ′ vs. T|_f_ temperature peak. The resulting Δ vs. 1/δ dependence is shown in Figure 4. We note that, initially, Δ increases monotonically with the dilution of the ferrofluid, from 0.11 at δ = 0.66 mg/mL (the as-prepared sample) to 0.188 at δ_c_ = 0.02 mg/mL. However, further diluting the ferrofluid has no effect on Δ. Indeed, Δ stays constant (within the margin of error) at ~0.185 for ferrofluid concentrations between 0.02 and 0.005 mg/mL. As discussed above, Δ has been shown to contain information on thermally driven superspin dynamics in the presence of interparticle dipolar interactions. In that context, our interpretation of the results shown in Figure 4 is the following. Above δ_c_, i.e., for 0.66 mg/mL > δ > 0.02 mg/mL, the interparticle dipolar interactions affect the superspin dynamics of the ensemble, as indicated by the increase in the value of Δ upon dilution. In addition, the observed smooth variation in Δ indicates that *the same* collective superspin relaxation and blocking–unblocking mechanism (most likely, the one described by the Vogel–Fulcher law in Equation (3)) operates throughout this high-concentration regime. Below δ_c_, however, Δ does not change upon dilution, which suggests that the interparticle interactions become negligible and the system behaves as an ideal magnetic nanoparticle ensemble where the superspins relax independently. If our interpretation is confirmed, this result is important for at least two reasons. First, because it provides an experimental procedure to determine the critical concentration, δ_c_, below which the dynamics of a magnetic nanoparticle ensemble is not affected by interparticle dipolar interactions. Second, because once the nanoparticle chemical composition and average size are known, it allows the design of systems of superspins that relax independently. The critical concentration δ_c_ is significant because, below this value, the superspin relaxation of any of the nanoparticles in the ensemble only depends on the temperature, T, and the energy barrier to magnetization reversal, E_B_, and is not affected by the magnetic fields from the adjacent nanoparticles. This has major implications for the functionality of high-density magnetic recording devices where each superspin represents one recording bit. Therefore, the next step is to confirm the non-interacting and individual spin relaxation nature of the Fe_3_O_4_/hexane ferrofluids at concentrations δ ≤ δ_c_. We achieved this by using ac susceptibility data and analysis based on the Néel–Brown activation law, which describes the temperature dependence of the relaxation time for an ideal ensemble of nanoparticles.

**Figure 5:**
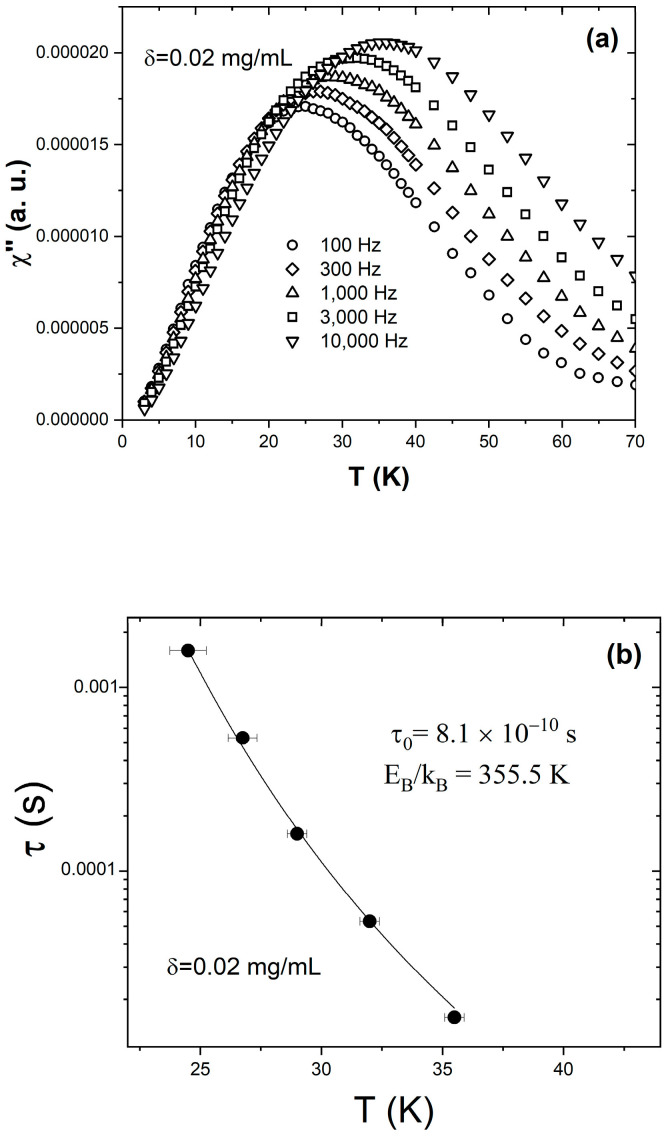
(**a**) Temperature dependence of the out-of-phase component of the ac susceptibility χ′ measured on the δ = 0.02 mg/mL sample at different frequencies: 100 Hz (circles), 300 Hz (diamonds), 1000 Hz (upright triangles), 3000 Hz (squares), and 10,000 Hz (inverted triangles). (**b**) Temperature dependence of the relaxation time obtained from the ac susceptibility data collected at different frequencies (filled symbols) and best fit to a Néel–Brown activation law (line).

Figure 5a shows the temperature dependence of the out-of-phase susceptibility χ″ measured from the δ = 0.02 mg/mL sample at different frequencies ranging from 100 Hz to 10,000 Hz. All the χ″ vs. T|_f_ curves peak at the blocking temperature, T_B_, where the superspin relaxation time is equal to the observation time $\tau =\frac{1}{2\pi f}$. Consequently, the peak shifts towards higher temperatures with the decrease in the observation time. The five solid symbols in Figure 5b represent the τ(T) dependence obtained from the ac susceptibility data discussed above. To verify if this behavior corresponds to a system of magnetic nanoparticles where the dipolar interactions are so weak that the superspins relax individually, we attempted to model the observed τ(T) dependence using the Néel–Brown equation $\tau \left(T\right)=\tau_{0}\exp\left(\frac{E_{B}}{k_{B}T}\right)$. The solid line represents the best fit, obtained upon the simultaneous variation in two parameters: the characteristic time τ_0_ and the reduced energy barrier to magnetization reversal E_B_/k_B_. The fit converges and attains low residuals, and yields τ_0_ = 8.1 × 10^−10^ s and E_B_/k_B_ = 355.5 K. We carried out similar measurements and analyses for the δ = 0.01 mg/mL and δ = 0.005 mg/mL samples, and found that the observed τ(T) dependence is also excellently described by the Néel–Brown model (Equation (1)). This is important, as it demonstrates that when diluted below the critical concentration, δ ≤ δ_c_, the Fe_3_O_4_/hexane ferrofluid behaves like an “ideal” nanoparticle ensemble, where the interparticle distances are large enough to render the dipolar interactions negligible. Based on a simple model that assumes that the 8 nm diameter Fe_3_O_4_ nanoparticles are uniformly distributed within the carrier fluid, we estimated the critical interparticle distance (that corresponds to the critical concentration δ_c_ = 0.02 mg/mL) to be d_c_ = 4.06 × 10^−7^ m. Increasing the interparticle distance above this value prevents the typical collective superspin relaxation—which is driven by interparticle magnetic interactions—and enables the superspins in the ensemble to flip individually as they do in isolated nanoparticles for which the Néel–Brown model was developed.

**Figure 6:**
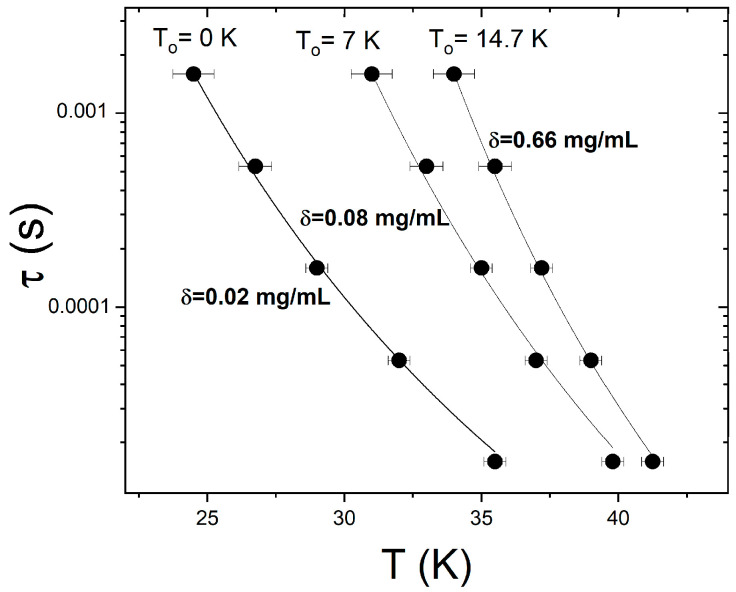
Temperature dependence of the relaxation time obtained from ac susceptibility data collected at different frequencies (filled symbols) and best fits to a Vogel–Fulcher activation law (lines) for three different concentrations: δ = 0.02 mg/mL, δ = 0.08 mg/mL, and δ = 0.66 mg/mL.

Finally, we used temperature-resolved ac susceptibility measurements carried out at different frequencies to investigate the superspin dynamics in the denser samples where the concentration is above the critical value, i.e., δ ≥ δ_c_. The solid symbols in Figure 6 show the temperature dependence of the relaxation time, τ(T), obtained from the aforementioned ac susceptibility data collected on the δ = 0.66, δ = 0.08, and δ = 0.02 mg/mL samples. The solid lines correspond to best fits of the Vogel–Fulcher activation law, $\tau \left(T\right)=\tau_{0}\exp\left[\frac{E_{B}}{k_{B}\left(T-T_{0}\right)}\right]$. 

This law describes the collective relaxation of the superspins in the presence of interparticle dipolar interactions and includes a parameter, T_0_, that describes the strength of these interactions. According to the Shtrikman and Wohlfarth model, T_0_ decreases with the decrease in the interparticle interactions. In our case, this corresponds to the decrease in the concentration upon dilution, so we expect T_0_ to decrease with the decrease in δ to between 0.66 and 0.02 mg/mL. Indeed, the fits in Figure 6 yield values of the dipolar interaction parameter that decrease upon the progressive dilution of the as-prepared ferrofluid: T_0_ = 14.7 K for δ = 0.66 mg/mL, T_0_ = 7 K for δ = 0.08 mg/mL, and T_0_ = 0 for δ = 0.02 mg/mL. It is worth noting that the analysis above further confirms the lack of interparticle dipolar interactions once the critical concentration of 0.02 is reached upon dilution. In addition, we find that the same model (based on Equation (3)) accurately describes the superspin dynamics throughout the entire higher concentration regime, 0.66 mg/mL > δ > 0.02 mg/mL, indicating that superparamagnetic blocking (and not superspin freezing) occurs in all these ferrofluids upon cooling below T_B_.

## 4. Summary

We studied the effect of interparticle dipolar interactions on the superspin relaxation of Fe_3_O_4_ magnetic nanoparticle ensembles of an average diameter <D> = 8 nm. We used Fe_3_O_4_/hexane ferrofluids of different concentrations, δ, ranging from 0.66 to 0.005 mg (Fe_3_O_4_)/mL (hexane) to control interparticle dipolar interaction strength. Our main finding was a critical concentration δ_c_ = 0.02 mg/mL that gives rise to two separate superspin dynamics regimes. Temperature-resolved ac susceptibility data collected at different frequency/observation times show that when δ ≤ δ_c_ the superspins relax individually as in an ideal ensemble of non-interacting magnetic nanoparticles. That was demonstrated by successful fits of the Néel–Brown activation law (Equation (1)) to the τ(T) data collected from the highly diluted samples, and further confirmed by the invariance of the relative peak temperature variation per frequency decade $∆=\frac{∆T}{T\cdot ∆log(f)}$ for δ ≤ δ_c_. The ability to experimentally determine the nanoparticle concentration below which the interparticle dipolar interactions do not have any effect on the superspin dynamics is important for several applications of magnetic nanoparticles. Indeed, the absence of such an effect allows a nanoparticle ensemble to function as a high-density magnetic recording medium where each superspin represents a separate recording bit that relaxes independently. For nanoparticle concentrations above δ_c_ (0.66 mg/mL > δ > 0.02 mg/mL), a different behavior was observed. Here, Δ exhibits a monotonic increase with the decrease in the ferrofluid concentration, indicating the presence interparticle dipolar interactions that influence the superspin dynamics. We modeled the superspin relaxation using a Vogel–Fulcher law (Equation (3)), which allowed us to quantitatively characterize the dipolar interaction strength throughout this regime.

## Data Availability

The data presented in this study are available on reasonable request from the corresponding author.
